# *International Journal of Molecular Science* 2017 Best Paper Award

**DOI:** 10.3390/ijms18112310

**Published:** 2017-11-02

**Authors:** International Journal of Molecular Science Editorial Office

**Affiliations:** MDPI AG, St. Alban-Anlage 66, 4052 Basel, Switzerland; ijms@mdpi.com

The Editors of the *International Journal of Molecular Sciences* have established the Best Paper Award to recognize the most outstanding articles published in the areas of molecular biology, molecular physics and chemistry that have been published in the *International Journal of Molecular Sciences*. The prizes have been awarded annually since 2012 [1,2,3,4,5].

We are pleased to announce the “*International Journal of Molecular Science* Best Paper Award” for 2017. Nominations, chosen from all papers published in 2016, were made by the Editorial Board. The awards are issued to reviews and research articles separately. Following a review process by the Editorial Board, three top-voted research articles and the three top-voted reviews as follows, in no particular order, have won “*International Journal of Molecular Science* Best Paper Award” for 2017:

## Research Article Award:

**A RNA–DNA Hybrid Aptamer for Nanoparticle-Based Prostate Tumor Targeted Drug Delivery**

John C. Leach, Andrew Wang, Kaiming Ye and Sha Jin

*Int. J. Mol. Sci.* **2016**, *17*(3), 380; doi:10.3390/ijms17030380

Available online: http://www.mdpi.com/1422-0067/17/3/380

**Figure 1:**
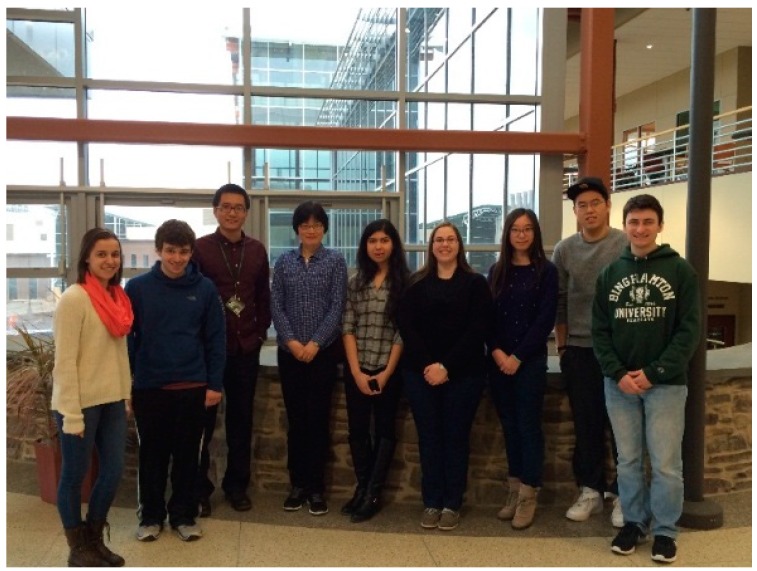
Dr. Sha Jin’s research group.

This research article reported the development of a new nanoscale drug delivery platform for cancer therapy. In this report, a newly designed DNA–RNA hybridized aptamer, designated as A10-3-J1, is able to recognize the extracellular domain of the prostate specific membrane antigen (PSMA). A super paramagnetic iron oxide nanoparticle conjugated with aptamer-doxorubicin (SPIO-Apt-Dox) has been fabricated and employed as a targeted drug delivery agent. The experimental results indicate that the aptamer anti-tumor agent inhibits nonspecific uptake of membrane-permeable doxorubicin to the non-target cells, leading to reduced untargeted cytotoxicity and endocytic uptake. In the meantime, it has specificity to targeted PSMA^+^ prostate cancer cells, resulting in the deaths of most of the cells. This report demonstrated that the drug delivery platform yields statistically significant effectiveness, being more cytotoxic to the targeted cells as opposed to the non-targeted cells (Figure 1).

**Relevance of MicroRNA200 Family and MicroRNA205 for Epithelial to Mesenchymal Transition and Clinical Outcome in Biliary Tract Cancer Patients**

Romana Urbas, Christian Mayr, Eckhard Klieser, Julia Fuereder, Doris Bach, Stefan Stättner, Florian Primavesi, Tarkan Jaeger, Stefanie Stanzer, Anna Lena Ress, Magdalena Löffelberger, Andrej Wagner, Frieder Berr, Markus Ritter, Martin Pichler, Daniel Neureiter and Tobias Kiesslich

*Int. J. Mol. Sci.* **2016**, *17*(12), 2053; doi:10.3390/ijms17122053

Available online: http://www.mdpi.com/1422-0067/17/12/2053

**Figure 2:**
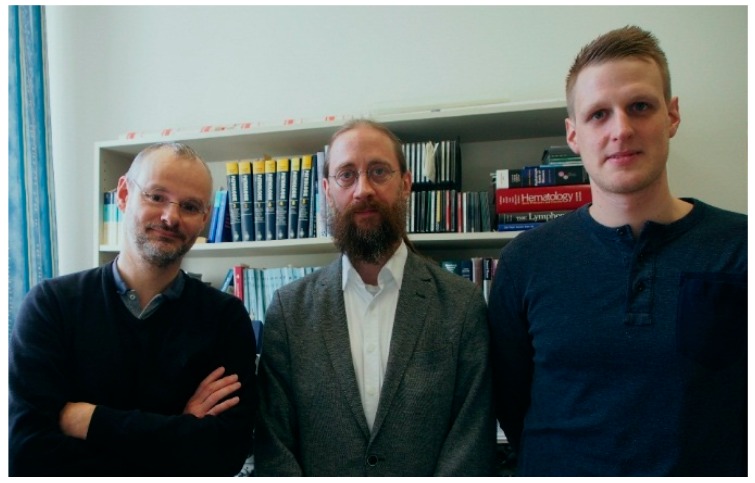
Dr. Daniel Neureiter’s research group.

Biliary tract cancer (BTC) is a very aggressive kind of cancer characterized by late diagnosis of disease, still limited therapeutic options and bad outcome. Hence, there is an urgent need for new therapeutic approaches and targets. A commonly observed histopathological characteristic of BTC is an intensive epithelial-to-mesenchyml transiton (EMT)—a key event in tumor progression und metastasis and potential reason for the dismal outcome of BTC. It is known that EMT is essentially regulated by microRNAs (miRs). Therefore, we started a comprehensive molecular-pathological in-vitro and in-situ expression investigation of the anti-EMT miR200 family (miR141, −200a/b/c, −429) and miR205 as well as of the EMT-related proteins E-Cadherin and Vimentin in a panel of BTC cell lines and clinical specimens. The results revealed a significant association of miR expression with (i) the expression patterns of E-Cadherin and Vimentin; (ii) clinicopathological characteristics; and (iii) survival data. The detailed statistical survival analysis could demonstrate that expression levels of the miR200 family members (but not miR205) as well as of E-Cadherin and Vimentin could serve as positive and negative prognostic markers in cases with BTC and possibly as therapeutic targets in the future (Figure 2).

**Comparative Study of Green Sub- and Supercritical Processes to Obtain Carnosic Acid and Carnosol-Enriched Rosemary Extracts with in Vitro Anti-Proliferative Activity on Colon Cancer Cells**

Andrea del Pilar Sánchez-Camargo, Virginia García-Cañas, Miguel Herrero, Alejandro Cifuentes and Elena Ibáñez

*Int. J. Mol. Sci.* **2016**, *17*(12), 2046; doi:10.3390/ijms17122046

Available online: http://www.mdpi.com/1422-0067/17/12/2046

**Figure 3:**
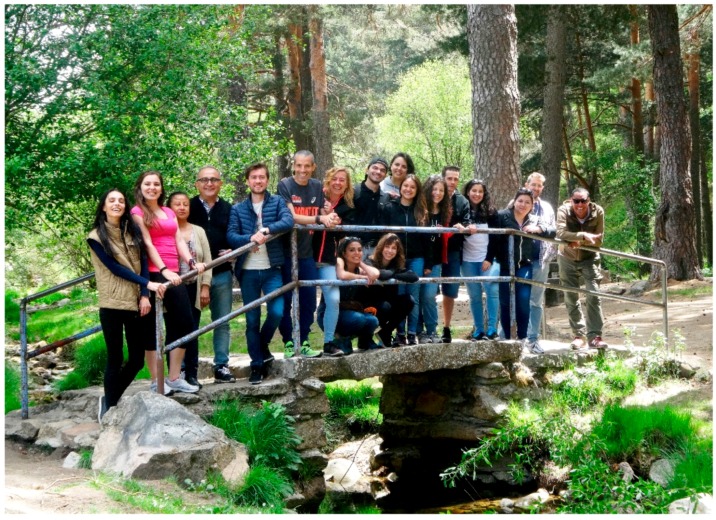
Dr. Elena Ibáñez’s research group.

In the last few years, our laboratory has been intensively working on the Foodomics evaluation of rosemary extracts with in-vitro antiproliferative activity against colon cancer cell lines. During our studies, two compounds were detected as the main cause of the observed bioactivity (carnosic acid and carnosol). In the present work, we evaluated the potentiality of four different green extraction processes to obtain enriched fractions of these two phenolic diterpenes; these processes were all based on the use of compressed fluids’ technologies, employing green solvents and following the most recent trend of intensifying or integrating processes. Moreover, an in-depth chemical characterization of the most active extracts (obtained combining pressurized liquid extraction and supercritical antisolvent fractionation, PLE + SAF) suggested that other compounds, such as rosmaridiphenol and safficinolide, could also play a role as additional contributors to the observed strong anti-proliferative activity of carnosic acid and carnosol enriched extracts (Figure 3).

## Review Paper Award:

**Telomeres, NAFLD and Chronic Liver Disease**

Benedetta Donati and Luca Valenti

*Int. J. Mol. Sci.* **2016**, *17*(3), 383; doi:10.3390/ijms17030383

Available online: http://www.mdpi.com/1422-0067/17/3/383

In the Review article entitled “Telomeres, NAFLD and Chronic Liver Disease”, we survey the literature supporting a role of telomeres homeostasis and Telomerase complex activity in the pathogenesis of non-alcoholic fatty liver disease (NAFLD), now the most frequent cause of liver damage worldwide, focusing on its link to the progression of chronic liver disease and cancer.

**Figure 4:**
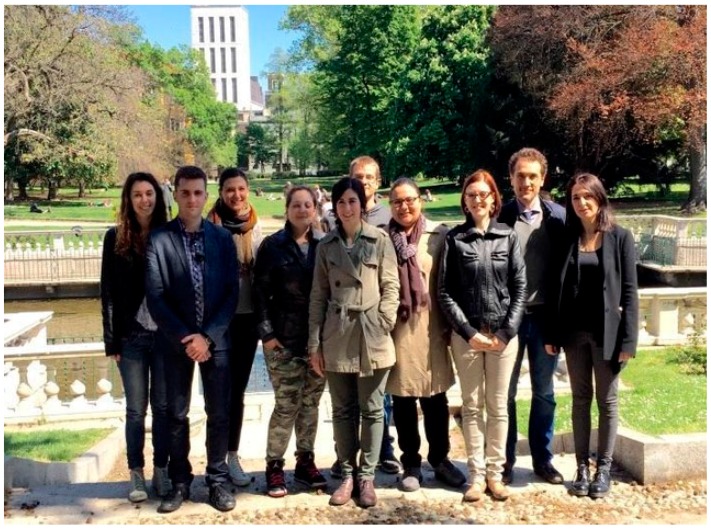
Dr. Luca Valenti’s research group.

We describe telomeres shortening as a genetic tract that can be inherited through generations predisposed to organ failure and often connected to the occurrence of mutations in Telomerase complex genes. Indeed, experimental evidence links telomere impairment to degenerative and chronic conditions such as fibrosis, defining telomere shortening, a hallmark of cirrhosis. Furthermore, we summarize the different mechanisms of telomeres re-elongation in the development of hepatocellular carcinoma (HCC) including Telomerase reverse transcriptase (TERT) reactivation, the increased expression of the other proteins involved in telomere regulation and alternative lengthening of telomere (ALT). We highlighted that TERT also influences liver carcinogenesis by other pathways independent of telomeres regulation. In conclusion, we propose a mechanism accounting for the predisposing effect of telomeres length deregulation and TERT germline mutations on the pathogenesis of NAFLD-HCC, a condition strongly influenced by genetic factors. Finally, we speculate on the possible use of drugs targeting the telomere machinery in clinical practice for the prevention and treatment of severe liver disease related to NAFLD and other causes of liver damage (Figure 4).

**The Functions of Metallothionein and ZIP and ZnT Transporters: An Overview and Perspective**

Tomoki Kimura and Taiho Kambe

*Int. J. Mol. Sci.* **2016**, *17*(3), 336; doi:10.3390/ijms17030336

Available online: http://www.mdpi.com/1422-0067/17/3/336

**Figure 5:**
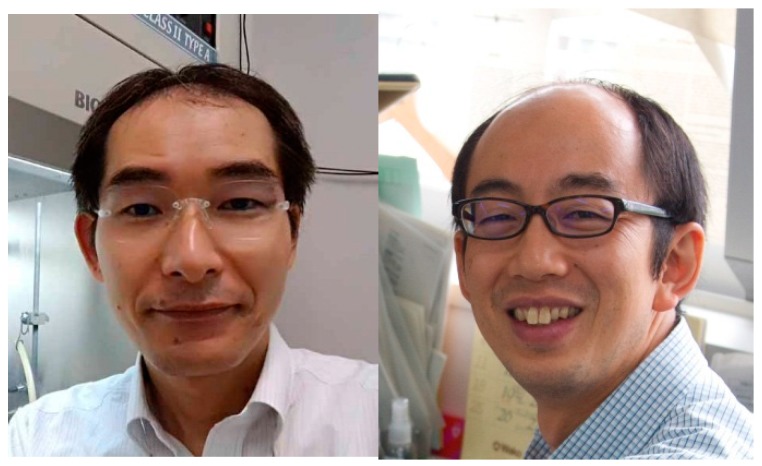
Dr. Tomoki Kimura (**left**) and Dr. Taiho Kambe (**right**).

The field of zinc metabolism and homeostasis has undergone a dramatic expansion. In recent decades, an increasing amount of evidence has uncovered critical roles of a number of proteins in zinc metabolism and homeostasis through influxing, chelating, sequestrating, coordinating, releasing, and effluxing zinc. Metallothioneins (MT) and Zrt- and Irt-like proteins (ZIP) and Zn transporters (ZnT) are the proteins primarily involved in these processes, and thus play crucial roles in maintaining the cellular zinc homeostasis. This review paper summarizes our understanding of the physiological and biological functions of MTs and ZIP and ZnT transporters focusing on recent progresses. We hope it provides a better understanding of zinc biology as well as an understanding of the roles of zinc in health and disease (Figure 5).

**Reactivities of Quinone Methides versus *o*-Quinones in Catecholamine Metabolism and Eumelanin Biosynthesis**

Manickam Sugumaran

*Int. J. Mol. Sci.* **2016**, *17*(9), 1576; doi:10.3390/ijms17091576

Available online: http://www.mdpi.com/1422-0067/17/9/1576

**Figure 6:**
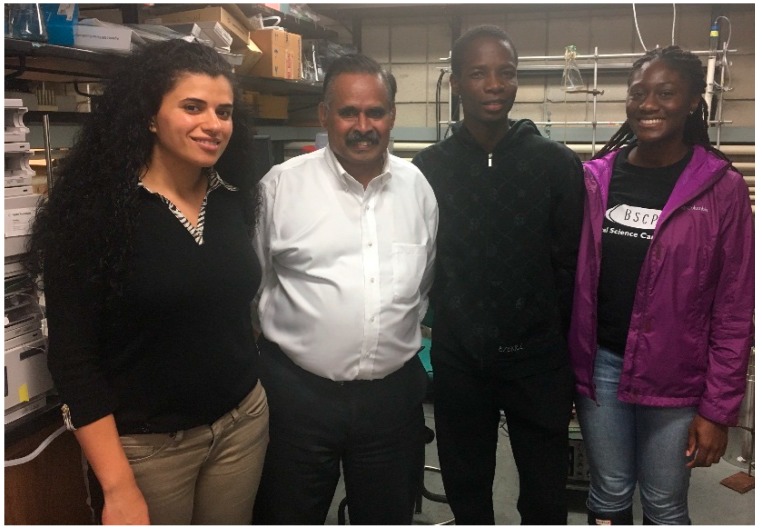
Hanine Barek, Manickam Sugumaran, Kenel Dufort, and Jessie M Ngandjui (from left to right).

Catecholamines form the building blocks of melanin pigment in animals and the exoskelatal pigment in insects. Tyrosine and dopa serve as the precursor for melanogenesis and a related process, called sclerotization in insects. My laboratory is actively involved in studying the metabolic fate of catecholamines and enzymes associated with the process. The overall aim of our research is to establish the importance of quinone methides in the biochemistry of catecholamine metabolism involved in melanin biosynthesis, insect cuticular sclerotization, and bioadhesive/antibacterial activities of tunicates and mussels. In this article, the detailed biochemistry of quinone methides formed during the biotransformation of different catecholamines is discussed. In melanin biosynthesis, quinone methides are generated as key intermediates of dopachrome conversion to dihydroxyindoles. In addition, they are also involved at the later stages of indole polymerization. In insect cuticular sclerotization, quinone methide derivatives such as *N*-acyldopamine quinone methides and quinone methide imine amides play a vital role in the crosslinking and hence hardening reactions. Finally, the biological activities of dopa/dehydrodopyl peptides found in numerous marine organisms are often exhibited through transient quinone methide intermediates. Our laboratory also identified three enzymes associated with quinone methide metabolism. Thus, this review highlights the importance of quinone methides as key transient intermediates of numerous catecholamine metabolisms (Figure 6).

We believe that these six exceptional papers are valuable contributions to the *International Journal of Molecular Science* and the scientific research field. On behalf of the *International Journal of Molecular Science* Editorial Board, we would like to congratulate these teams for their excellent work. In recognition of their accomplishment, they will receive the privilege of publishing an additional research article or review paper free of charge in open access format in the *International Journal of Molecular Science*, after the usual peer-review procedure.

We would like to take this opportunity to thank all the nominated research groups of the above exceptional papers for their contributions to the *International Journal of Molecular Science*, and thank the *International Journal of Molecular Science* Editorial Board for voting and helping with this “Best Paper Award”.

The Editorial Board and Editorial Staff at the *International Journal of Molecular Science* is committed to meeting the needs of the molecular research community by providing useful and timely reviews of all manuscripts submitted, and providing an open access journal for your results. Please consider submitting your work to the *International Journal of Molecular Science*, and we look forward to announcing your paper as an *International Journal of Molecular Science* Best Paper in the future.

## Prize Awarding Committee

*International Journal of Molecular Science* Editorial Board.
